# Chronic HIV infection induces transcriptional and functional reprogramming of innate immune cells

**DOI:** 10.1172/jci.insight.145928

**Published:** 2021-04-08

**Authors:** Wouter A. van der Heijden, Lisa Van de Wijer, Farid Keramati, Wim Trypsteen, Sofie Rutsaert, Rob ter Horst, Martin Jaeger, Hans J.P.M. Koenen, Hendrik G. Stunnenberg, Irma Joosten, Paul E. Verweij, Jan van Lunzen, Charles A. Dinarello, Leo A.B. Joosten, Linos Vandekerckhove, Mihai G. Netea, André J.A.M. van der Ven, Quirijn de Mast

**Affiliations:** 1 Department of Internal Medicine, Radboud Center for Infectious Diseases, Radboud University Medical Center, Nijmegen, Netherlands.; 2 Department of Molecular Biology, Faculty of Science, Radboud University, Nijmegen, Netherlands.; 3 HIV Cure Research Center, Department of Internal Medicine and Pediatrics, Faculty of Medicine and Health Sciences, Ghent University and Ghent University Hospital, Ghent, Belgium.; 4 Department of Laboratory Medicine, Laboratory for Medical Immunology, Radboud University Medical Center, Nijmegen, Netherlands.; 5 Department of Medical Microbiology, Radboud University Medical Center and Center of Expertise in Mycology Radboudumc/CWZ, Nijmegen, Netherlands.; 6 ViiV Healthcare, Brentford, United Kingdom.; 7 Department of Medicine and Immunology, University of Colorado School of Medicine, Aurora, Colorado, USA.; 8 Department for Genomics & Immunoregulation, Life and Medical Sciences Institute, University of Bonn, Bonn, Germany.

**Keywords:** AIDS/HIV, Immunology, Innate immunity, Monocytes, Transcription

## Abstract

Chronic inflammation and immune dysfunction play a key role in the development of non-AIDS–related comorbidities. The aim of our study was to characterize the functional phenotype of immune cells in people living with HIV (PLHIV). We enrolled a cross-sectional cohort study of PLHIV on stable antiretroviral therapy and healthy controls. We assessed ex vivo cytokine production capacity and transcriptomics of monocytes and T cells upon bacterial, fungal, and viral stimulation. PLHIV exhibited an exacerbated proinflammatory profile in monocyte-derived cytokines, but not in lymphocyte-derived cytokines. Particularly, the production of the IL-1β to imiquimod, *E*. *coli* LPS, and *Mycobacterium tuberculosis* was increased, and this production correlated with plasma concentrations of high-sensitivity C-reactive protein and soluble CD14. This increase in monocyte responsiveness remained stable over time in subsequent blood sampling after more than 1 year. Transcriptome analyses confirmed priming of the monocyte IL-1β pathway, consistent with a monocyte-trained immunity phenotype. Increased plasma concentrations of β-glucan, a well-known inducer of trained immunity, were associated with increased innate cytokine responses. Monocytes of PLHIV exhibited a sustained proinflammatory immune phenotype with priming of the IL-1β pathway. Training of the innate immune system in PLHIV likely plays a role in long-term HIV complications and provides a promising therapeutic target for inflammation-related comorbidities.

## Graphical abstract

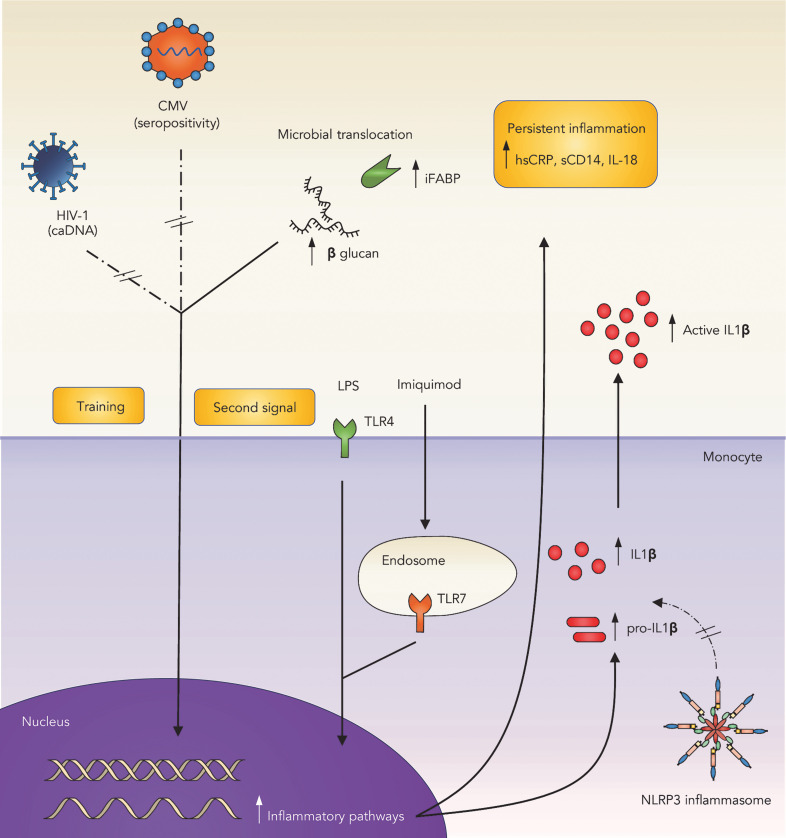

## Introduction

Persistent inflammation and immune dysfunction play an important role in the development of non-AIDS–related comorbidities in people living with HIV (PLHIV). Those include cardiovascular disease (CVD), neurocognitive dysfunction, and cancer (1–4). Reducing inflammation is considered an attractive therapeutic target to reduce the burden of non-AIDS–related events. However, immune dysfunction and persistent inflammation in PLHIV are complex processes that are still incompletely understood. These involve changes in the adaptive immune system, including dysfunction and senescence of T cell lymphocytes (5), as well as changes in the innate immune system. Mediators of the latter, including soluble CD14 (sCD14), sCD163, and high-sensitivity C-reactive protein (hsCRP), are indicative of persistent immune activation and are associated with non-AIDS–related events (6–8).

Ongoing exposure to microbial products, for example, by CMV, continuing HIV replication, or microbial translocation, may be one of the drivers of persistent inflammation (2, 9–12). Increased inflammation can also result from a functional adaptation of innate immune cells induced by epigenetic reprogramming, a process termed “trained immunity” (13–16). Trained immunity is recognized to play an important beneficial role in host defense processes, but it may also contribute to conditions like atherosclerosis or type 2 diabetes mellitus (17, 18). Whether trained immunity contributes to persistent inflammation in PLHIV is currently unknown.

The Human Functional Genomics Project (HFGP) aims to investigate variation in the immune responses (19). Studies in HFGP cohorts in healthy individuals have yielded different novel insights in the genetic and nongenetic regulation of inflammatory cytokine responses in response to microbial ligands (20–23). We established a HFGP cohort of Dutch PLHIV, and in this cohort we describe alterations in the function and phenotype of PBMCs by using an integrative approach of functional and transcriptional analyses. We also explored underlying mechanisms of the inflammatory monocyte profile, including the possible contribution of exposure to microbial translocation, CMV seropositivity, and HIV reservoir size.

## Results

### Characteristics.

A total of 211 PLHIV on combination antiretroviral therapy (cART) and 56 HIV-uninfected controls were included in the analyses. Baseline characteristics are shown in Table 1. Compared with the healthy controls, PLHIV were more often men (91.5% vs. 60.7%, *P* < 0.001) and of older age (median [IQR] age of 52.5 [13.2] vs. 30.0 [27.1] years, *P* < 0.0001). The median (IQR) duration of cART use in PLHIV was 6.6 years (4–12 years), and 89% were virally suppressed (HIV viral copies ≤ 50 copies/mL) for longer than 1 year.

### Circulating inflammatory markers and innate cytokine responses.

**Figure 1:**
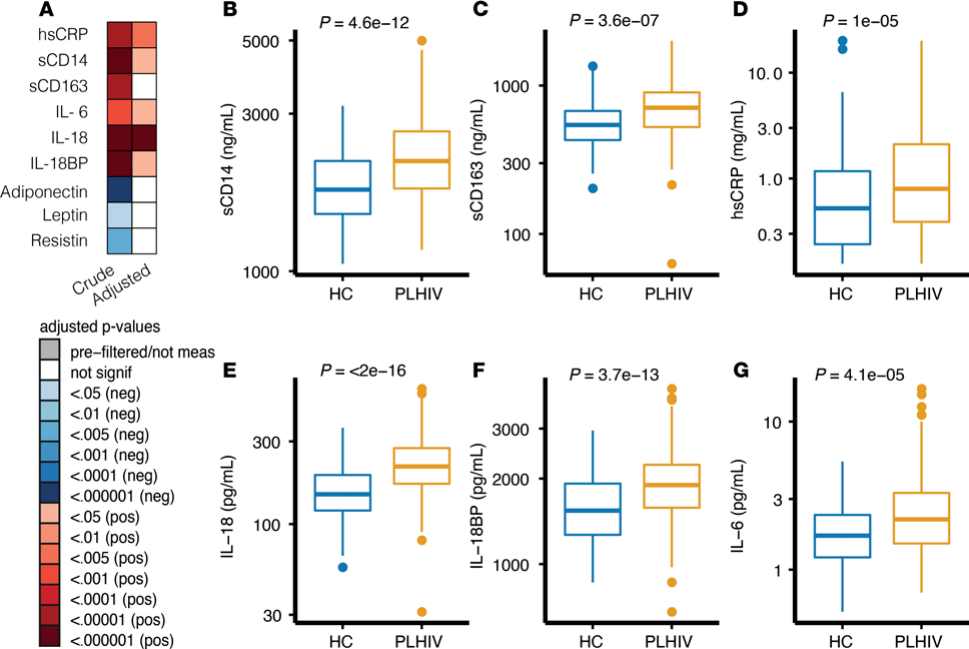
Circulating factors in PLHIV versus uninfected HCs. (**A**) Circulating factors in PLHIV and uninfected controls. Crude model is linear regression after inverse rank-based transformation. Adjusted model included age, sex, and seasonality as covariates. Red depicts the marker is significantly increased in PLHIV; blue depicts the marker is decreased in PLHIV compared with HCs. All *P* values are FDR corrected (Benjamini-Hochberg method). (**B**–**G**) Box plots depicting circulating factors of inflammation stratified by cohort; PLHIV (blue), uninfected controls (yellow). *P* values are calculated using 2-tailed Student’s *t* test. Box plots are depicted according to Tukey; median (line), IQR (edge of box plot), range (whiskers), and outliers 3 times IQR are depicted as dots. All plots include data from PLHIV (*n* = 211) and HCs (*n* = 56). PLHIV, people living with HIV; HCs, healthy controls; hsCRP, high-sensitivity C-reactive protein; sCD14, soluble CD14; sCD163, soluble CD163; IL-18BP, IL-18–binding protein.

We first measured concentrations of circulating inflammatory markers. Compared with healthy controls, PLHIV had significantly higher concentrations of sCD14, hsCRP, IL‑18, IL-18–binding protein (IL-18BP), and IL-6 (Figure 1, A–G). These differences remained after adjusting for age, sex, BMI, or seasonality (Figure 1A). Concentrations of adipokines were similar between groups after correction (Figure 1A).

**Figure 2:**
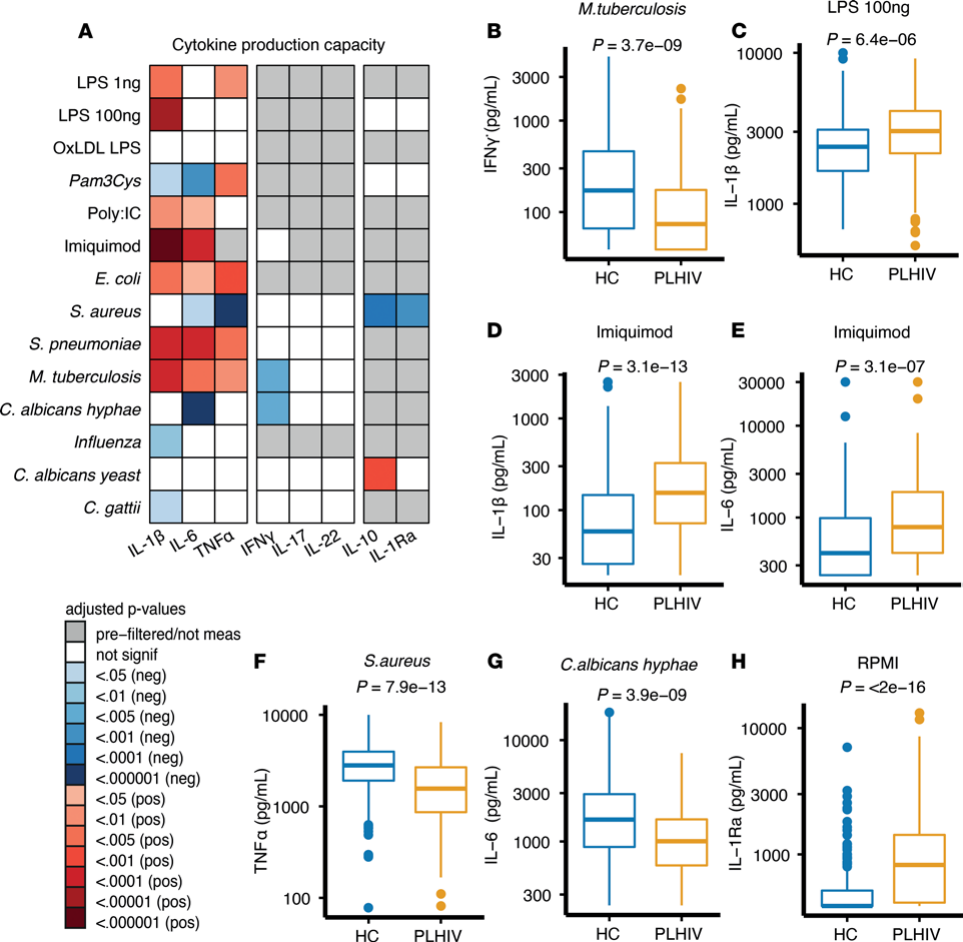
Cytokine production capacity in PLHIV versus uninfected HCs. (**A**) Ex vivo cytokine production capacity between PLHIV and HCs after 24 hours (in case of IL-1β, TNF-α, IL-6, IL-10, and IL-1Ra) and 7 days stimulation (IL-22, IL-17, and IFN-γ). FDR-corrected (Benjamini-Hochberg method) *P* values are depicted from an adjusted model that included age, sex, seasonality as covariates. Red depicts a significantly higher cytokine production capacity, and blue depicts a lower cytokine production capacity in PLHIV compared with HCs. (**B**–**H**) Box plots depicting ex vivo cytokine production capacity stratified by cohort. PLHIV depicted in blue and uninfected controls in yellow. *P* values, depicted in box plots, were calculated using 2-tailed Student’s *t* test after log transformation. All box plots are depicted according to Tukey; median (line), IQR (edge of box plot), range (whiskers), and outliers 3 times IQR are depicted as dots. All plots include data from PLHIV (*n* = 211) and HCs (*n* = 56). PLHIV, people living with HIV; HCs, healthy controls; oxLDL, oxidized LDL; Pam3Cys, synthetic TLR2 ligand; Poly IC, TLR3 ligand; imiquimod, TLR7 ligand.

Next, we analyzed functional changes in the innate and adaptive immune response. PBMCs were incubated with 12 stimuli (4 bacterial, 3 fungal, 1 viral, and 6 TLR ligands), followed by measurement of different monocyte-derived (IL-6, IL-1β, and TNF-α) and lymphocyte-derived (IL-17, IL-22, and IFN-γ) proinflammatory cytokines, as well as antiinflammatory cytokines (IL-10 and IL-1 receptor antagonist, IL-1Ra) in the supernatant. We observed markedly increased monocyte-derived cytokine responses in PLHIV, especially IL-1β responses upon stimulation with LPS (TLR4), imiquimod (TLR7), and *Mycobacterium tuberculosis* (Figure 2, A, C, and D). Production of TNF-α and IL-6 was also increased in PLHIV (Figure 2, A and E), except for lower production in response to *Staphylococcus aureus* (TNF-α) and *Candida albicans* (IL-6; Figure 1, F and G). This increased proinflammatory cytokine response in PLHIV was not associated with a concurrent increase in the antiinflammatory cytokines IL-1Ra or IL-10. However, the release of IL-1Ra by unstimulated cells was higher in PLHIV (Figure 2H), which is consistent with increased basal IL-1β production (Figure 2A). The number of monocytes in the isolated PBMCs (Supplemental Figure 1, A and B; supplemental material available online with this article; https://doi.org/10.1172/jci.insight.145928DS1) may influence cytokine production, but adjusting for the monocyte fraction in PBMCs did not significantly alter the outcome (Supplemental Figure 1C; T cell–derived cytokine responses were generally lower for most stimuli in the PLHIV cohort; Supplemental Figure 2). These differences were abrogated after adjustment for age, sex, and seasonality (Figure 1A), except for IFN-γ responses to *M*. *tuberculosis* and *C. albicans hyphae* (Figure 2, A and B). There were no associations between cytokine production capacity and smoking or different HIV-specific traits, including recent CD4 cell count, CD4/CD8 cell ratio, CD4 nadir, cART regimen, or comedication (metformin, statins, or aspirin) (Supplemental Figure 3).

**Figure 3:**
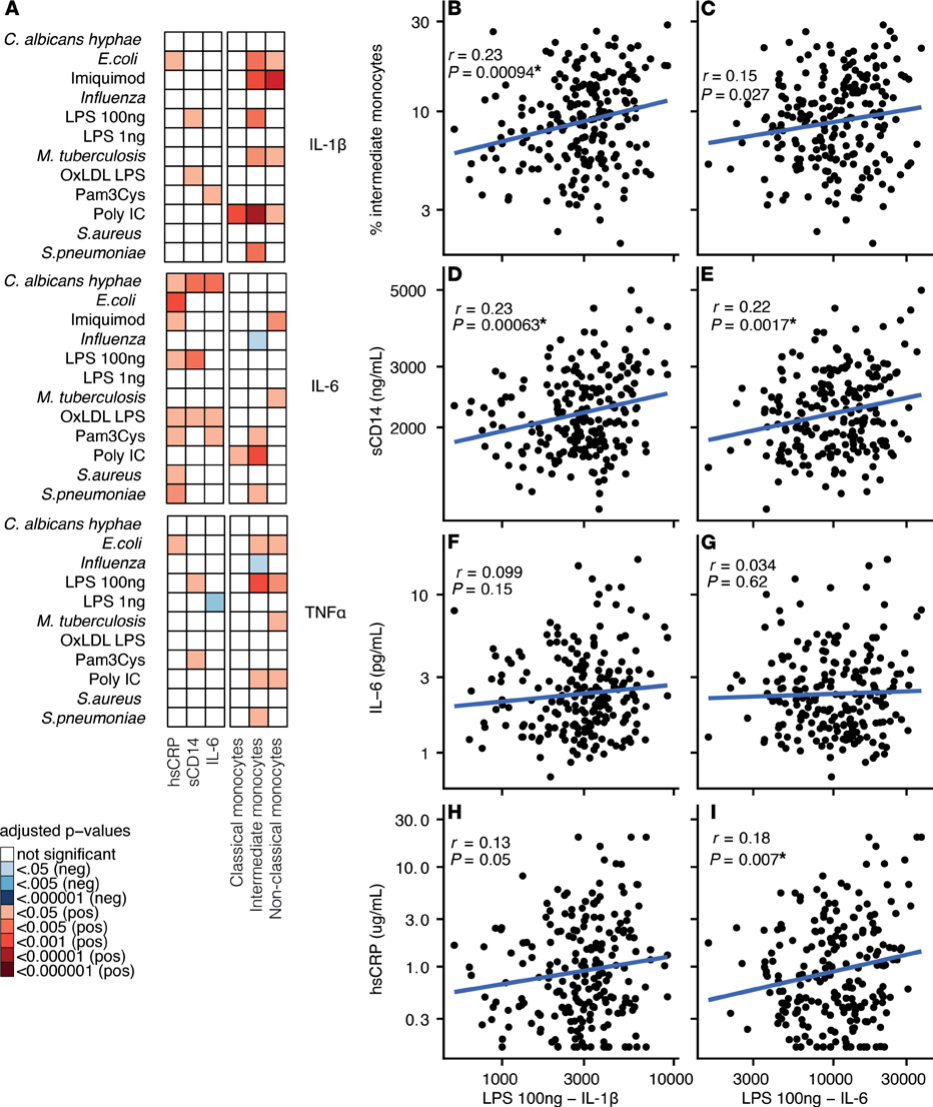
Cytokine production capacity versus circulating factors. (**A**) Correlation plot with adjusted *P* value shown after correcting for age, sex, and seasonality as covariates. Red depicts a significant positive correlation and blue depicts a negative correlation within PLHIV. All *P* values were FDR corrected per circulating factor. (**B**–**I**) Correlation plots without cofactor adjustment. Pearson’s coefficient (*r*) with *P* value after log transformation is shown. All plots include data from PLHIV (*n* = 211) and HCs (*n* = 56). PLHIV, people living with HIV; HCs, healthy controls; oxLDL, oxidized LDL; Pam3Cys, synthetic TLR2 ligand; Poly IC, TLR3 ligand; imiquimod, TLR7 ligand; hsCRP, high-sensitivity C-reactive protein; sCD14, soluble CD14; sCD163, soluble CD163; IL-18BP, IL-18–binding protein. *P < 0.05 after FDR correction.

Next, we assessed associations between innate cytokine responses, circulating inflammatory markers, and monocyte phenotypes (Figure 3, A–I). Increased ex vivo cytokine responses were associated with persistent inflammation. For example, we found positive associations between IL-6 responses and circulating hsCRP (Figure 3, A and I) and between LPS-induced IL-1β responses and plasma sCD14 and IL-18BP (Figure 3, A and D). In addition, nonclassical and intermediate monocyte subsets (Supplemental Figure 4, A–F), which are considered proinflammatory monocyte phenotypes, correlated with higher IL-1β and IL-6 production after LPS or imiquimod, respectively (intermediate monocytes vs. LPS-induced IL-1β *r* = 0.23, *P* < 0.001; Figure 3, A–C) (24). Taken together, we show that PLHIV have an altered innate immune profile with markedly elevated monocyte cytokine responses, particularly IL-1β, which correlates with blood biomarkers of persistent inflammation.

### Increased IL-1β production capacity of PLHIV was stable over time.

**Figure 4:**
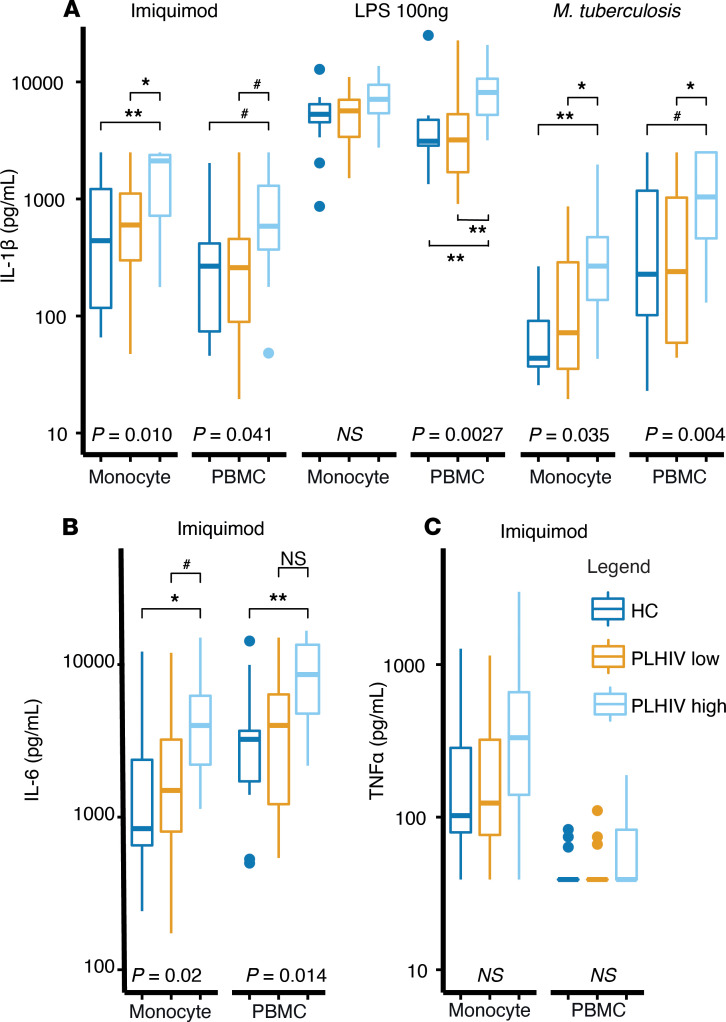
Ex vivo cytokine production capacity of monocytes in a validation cohort. (**A**) IL-1β production upon 24 hours’ stimulation with imiquimod (1 μg/mL), LPS (100 ng/mL), or *M*. *tuberculosis* (1 μg/mL) in PBMCs or monocyte-only culture (magnetic beads CD14^+^ isolation). (**B**) IL-6 production upon imiquimod stimulation. (**C**) TNF-α production upon imiquimod stimulation. All data are stratified by HCs (*n* = 14), PLHIV low initial IL-1β–producers (*n* = 13), and PLHIV high IL-1β–producers (*n* = 15). All box plots are depicted according to Tukey; median (line), IQR (edge of box plot), range (whiskers), and outliers 3 times IQR (dots). A Kruskal-Wallis test was performed with a post hoc testing when the *P* value was below 0.05. Post hoc testing was performed by using a Mann-Whitney *U* test on the comparison PLHIV high vs. HCs and PLHIV high vs. PLHIV low. A Bonferroni’s multiple-testing correction was used by setting the significance level at *P* < 0.025 for post hoc analysis. *P < 0.025, **P < 0.001, ^#^*P* < 0.05 (above multiple-testing threshold), ns, nonsignificant. PLHIV, people living with HIV; HCs, healthy controls.

We then assessed the longevity of the elevated monocyte-derived cytokine responses. We resampled 28 PLHIV, men older than 45 years of age, after more than 1 year and stratified them on the basis of their initial IL-1β production capacity in a group of low IL-1β–producers (lowest quartile) and high IL-1β–producers (highest quartile). Fourteen age- and sex-matched uninfected controls were enrolled concurrently (Supplemental Table 1). Upon resampling, those assigned to the high IL-1β–producer group still exhibited higher IL-1β and IL-6 production compared with participants in the low IL-1β–producer group and matched healthy controls, suggesting that the enhanced monocyte responsiveness was stable over time (Figure 4, A and B). TNF-α followed a similar but nonsignificant pattern (*P* = 0.075; Figure 4C). To examine if the presence of T lymphocytes affected the outcome, we isolated monocytes using magnetic beads (purity > 95%; Supplemental Figure 4G) and repeated the stimulation experiments. We found that IL-1β production remained increased, suggesting that the elevated monocyte-derived cytokine response was due to an enhanced functional state of the monocytes themselves.

### Enhanced proinflammatory functional state of monocytes.

**Figure 5:**
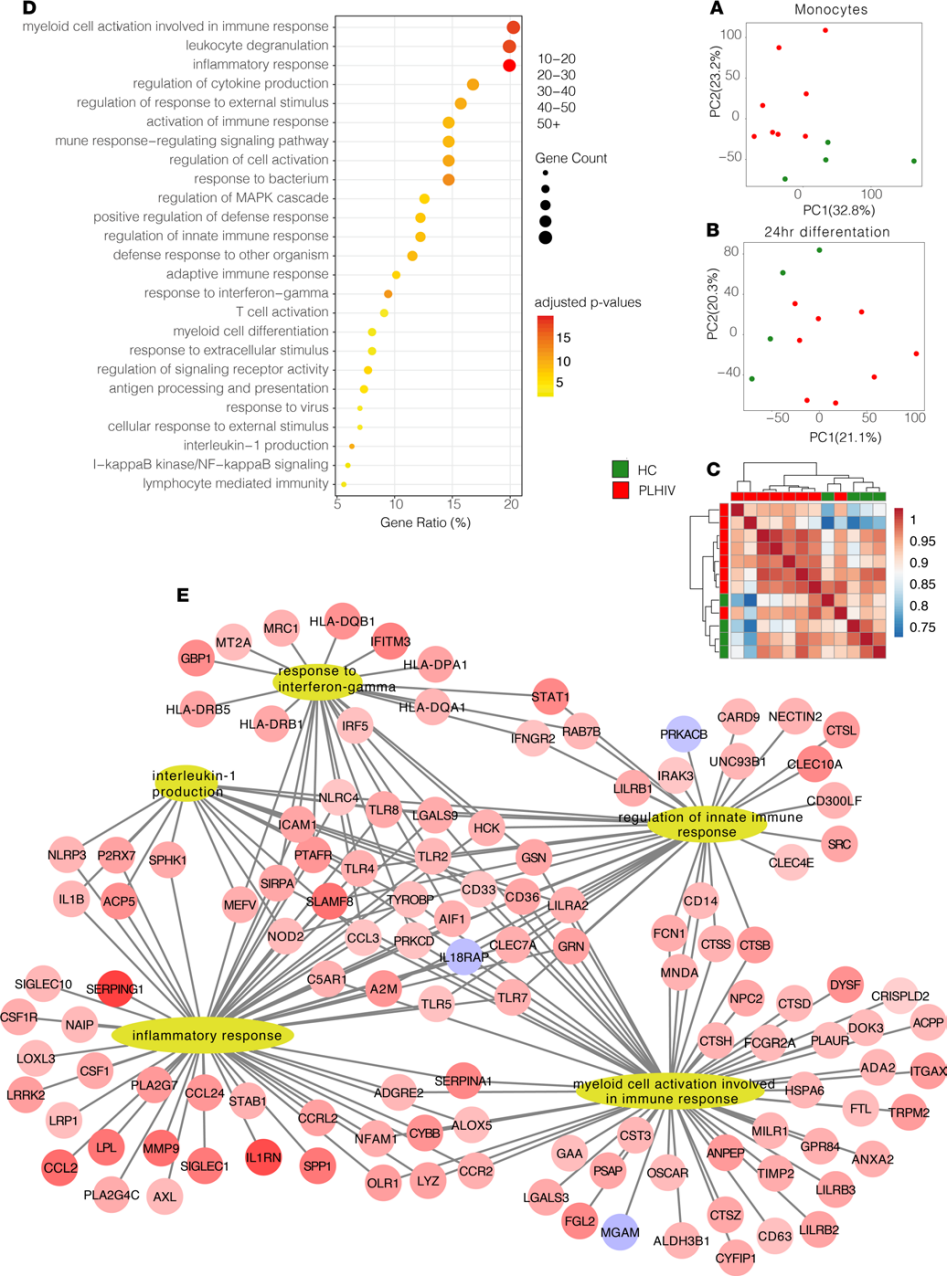
Transcriptome analysis of monocytes. (**A**) PCA plot of transcriptome of monocytes from PLHIV and HCs directly after isolation. (**B**) PCA plot of transcriptome of monocytes from PLHIV and HCs after 24 hours’ macrophage differentiation in medium only. (**C**) Hierarchical clustering plot (PLHIV vs. HCs). (**D**) Gene ontology of differentially expressed genes (PLHIV vs. HCs) including adjusted *P* value and gene count. (**E**) Top pathways of gene ontology plot after macrophage differentiation (gene ontology interaction terms). PLHIV, *n* = 8; HC, *n* = 4. PCA, principal component analysis; PLHIV, people living with HIV; HCs, healthy controls.

Based on this enhanced functional state, we postulated that a trained immunity functional phenotype (13, 14) contributes to the enhanced monocyte responsiveness in PLHIV. Hence, we isolated monocytes from 8 PLHIV and 4 age- and sex-matched uninfected controls and analyzed their transcriptomes by RNA-Seq. Using principal component analysis, we observed clustering of the PLHIV and controls (Figure 5A). This clustering was even more pronounced when monocytes were differentiated toward macrophage phenotype by culture for 24 hours in serum-free medium (RPMI; Figure 5B) and was also observed using hierarchical clustering (Figure 5C). Gene ontology analysis of 303 upregulated and 30 downregulated genes (Supplemental Table 5) showed that proinflammatory pathways were upregulated in PLHIV, including the “inflammatory response,” “regulation of innate immune response,” and “IL-1β production” pathways (Figure 5, D and E). Among the upregulated genes in these pathways were intracellular signaling proteins and inflammasome-related molecules (e.g., *NLRP3*, *STAT1*), cytokines (e.g., *IL1B*, *CCL2*, *MMP9*, *IL1RN*), and pattern recognition receptors (e.g., *TLR2*, *TLR4*, *TLR7*, *NOD2*), underlining the broad upregulation of inflammatory pathways in PLHIV on long-term cART.

### Enhanced IL-1β gene expression in monocytes of PLHIV.

**Figure 6:**
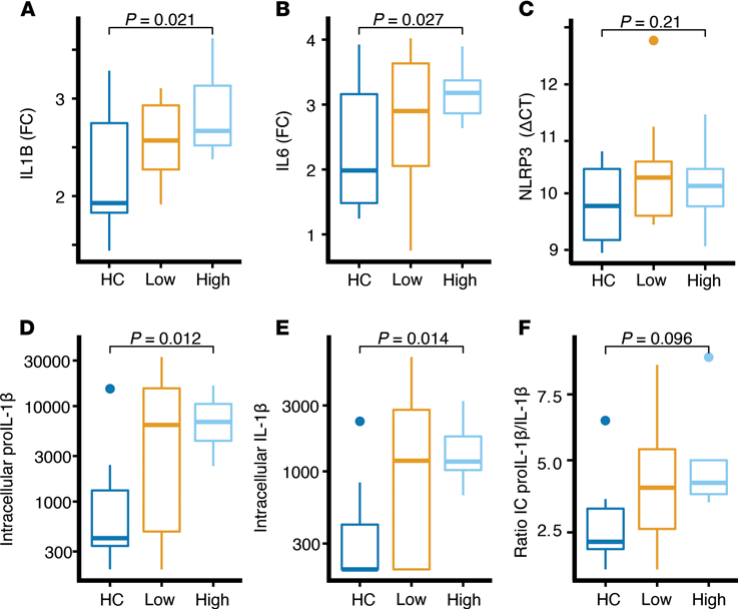
*IL1B* gene expression and intracellular pro–IL-1β. (**A**) *IL1B* gene expression after imiquimod 1 μg/mL stimulation depicted as FC from medium (RPMI). (**B**) *IL6* gene expression after imiquimod stimulation depicted as FC from medium. (**C**) *NLRP3* gene expression (by ΔCT) in RPMI. (**D**) Intracellular levels of pro–IL-1β protein after imiquimod stimulation. (**E**) Intracellular mature IL-1β protein after imiquimod stimulation. (**F**) Ratio of intracellular pro–IL-1β vs. IL-1β after imiquimod stimulation protein. All data are stratified by HCs (*n* = 14), PLHIV low initial IL-1β–producers (*n* = 13), and PLHIV high IL-1β–producers (*n* = 15). All box plots are depicted according to Tukey; median (line), IQR (edge of box plot), range (whiskers), and outliers 3 times IQR are depicted as dots. *P* values were calculated by 2-way ANOVA and subsequently by pair-wise 2-tailed Student’s *t* test. FC, fold change; PLHIV, people living with HIV; HCs, healthy controls.

The release of active IL‑1β from blood monocytes involves a priming signal that is mostly transcriptionally driven, which results in the synthesis of the IL‑1β precursor (pro–IL-1β). This precursor is subsequently processed via caspase-1 into active IL-1β (25). Using real-time quantitative PCR (RT-qPCR) on monocytes stimulated with IMQ, we found that the IL1B (gene encoding for pro–IL-1β) RNA expression was higher in monocytes from PLHIV compared with controls (Figure 6A; *P* = 0.021). *IL6* mRNA followed a similar trend (Figure 6B; *P* = 0.027), whereas *TNFA* expression did not (data not shown; *P* = 0.74). Additionally, we measured intracellular pro–IL-1β and active IL-1β protein by ELISA, and both were significantly elevated in PLHIV (Figure 6, D and E). The inflammasome promotes the proteolytic cleavage of pro–IL-1β into active IL-1β, resulting in reduced pro–IL-1β/active IL-1β ratios in situations with increased caspase-1 activity (25). Instead, we observed a trend toward an increased ratio of intracellular pro–IL-1β to active IL-1β (Figure 6F) in the high IL-1β–producing PLHIV and no differences in levels of NLRP3 expression (Figure 6C). Together, these results demonstrate that the enhanced IL-1β transcription, rather than increased processing through caspase-1, was the biological process that primarily drove the increased IL-1β production in PLHIV.

### β-Glucan exposure induced a proinflammatory monocyte phenotype.

**Figure 7:**
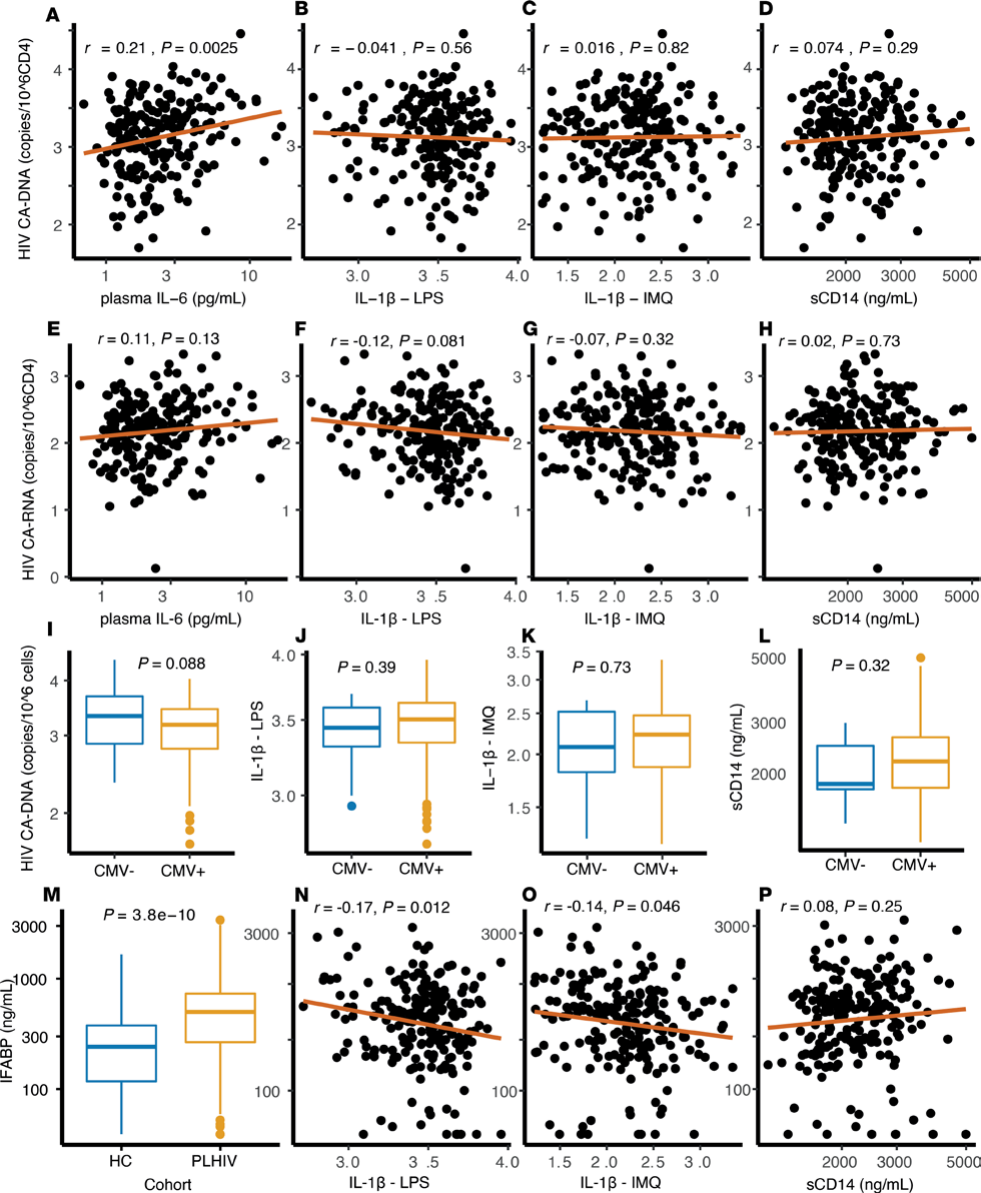
Parameters of HIV reservoir, CMV seropositivity, and microbial integrity. (**A**–**D**) Log10-transformed CA-DNA in CD4^+^ cells correlation with (**A**) circulating IL-6 levels, (**B**) IL-1β production after LPS (100 ng/mL) stimulation, (**C**) IL-1β production after IMQ (1 μg/mL) stimulation, and (**D**) soluble CD14 plasma concentration. (**E**–**H**) Log10-transformed CA-RNA in CD4^+^ cells correlation with (**E**) circulating IL-6 levels, (**F**) IL-1β production after LPS stimulation, (**G**) IL-1β production after imiquimod stimulation, and (**H**) sCD14 plasma concentration. (**I**–**L**) Stratified by CMV seropositivity: (**I**) log10-transformed CA-DNA in CD4^+^ cells, (**J**) IL-1β production after LPS stimulation, (**K**) IL-1β production after imiquimod stimulation, and (**L**) sCD14 plasma concentration. (**M**) Plasma iFABP, a marker of intestinal integrity, between HCs (*n* = 56) and PLHIV (*n* = 211). (**N**–**P**) Plasma iFABP concentration correlation with (**N**) IL-1β production after LPS stimulation, (**O**) IL-1β production after imiquimod, and (**P**) sCD14 plasma concentration. All box plots are depicted according to Tukey; median (line), IQR (edge of box plot), range (whiskers), and outliers 3 times IQR (dots). Pearson’s coefficient (*r*) after log transformation is shown in correlation plots. Data in box plots are analyzed using 2-tailed Student’s *t* test after log10 transformation. All plots depict data from PLHIV only (*n* = 211) unless otherwise stated. CA-DNA, cell-associated HIV-1 DNA; CA-RNA, cell-associated HIV-1 RNA; IMQ, imiquimod; iFABP, intestinal fatty acid–binding protein; PLHIV, people living with HIV; HCs, healthy controls.

We next examined possible pathways underlying the proinflammatory monocyte phenotype in PLHIV. Persistent exposure to microbial ligands derived from gut microbes, CMV, or HIV itself has been suggested to play a role in the persistent inflammation in PLHIV (2, 9–12). We postulated that these factors also play a role in the proinflammatory monocyte phenotype. We, therefore, first assessed whether innate cytokine responses were associated with parameters of the HIV viral reservoir. The HIV reservoir was assessed by analyzing CD4^+^ cell–associated HIV-1 DNA (CA-DNA) and CA-RNA. In virally suppressed patients, the CA-DNA roughly equals the integrated HIV-1 DNA, being replication competent or not (26), whereas CA-RNA is associated with recent HIV-1 transcriptional activity and serves as a proxy for the active proviral reservoir (27). Although CA-DNA levels in CD4^+^ T cells were associated with plasma IL-6 concentrations (Figure 7A), there was no association between IL-1β cytokine responses (Supplemental Figure 5) or plasma sCD14 concentrations and HIV-1 CA-DNA or CA-RNA levels in our cohort (Figure 7, B–H).

Next, we assessed associations between cytokine responses and CMV seropositivity. In total, 198 of 211 (93.8%) participants were CMV seropositive. There were no differences in IL-1β cytokine responses, sCD14 concentrations, or HIV-1 CA-DNA between CMV-seropositive versus -seronegative individuals (Figure 7, I–L, and Supplemental Figure 5).

Another source of chronic microbial ligand exposure in PLHIV is increased microbial translocation (9). Plasma concentrations of intestinal fatty acid–binding protein (iFABP), a marker for microbial translocation, were significantly increased in PLHIV compared with controls (Figure 7M). We found a modest negative correlation of iFABP concentrations with IL-1β cytokine responses (Figure 7, N and O) but no correlation with sCD14 concentrations (Figure 7P).

**Figure 8:**
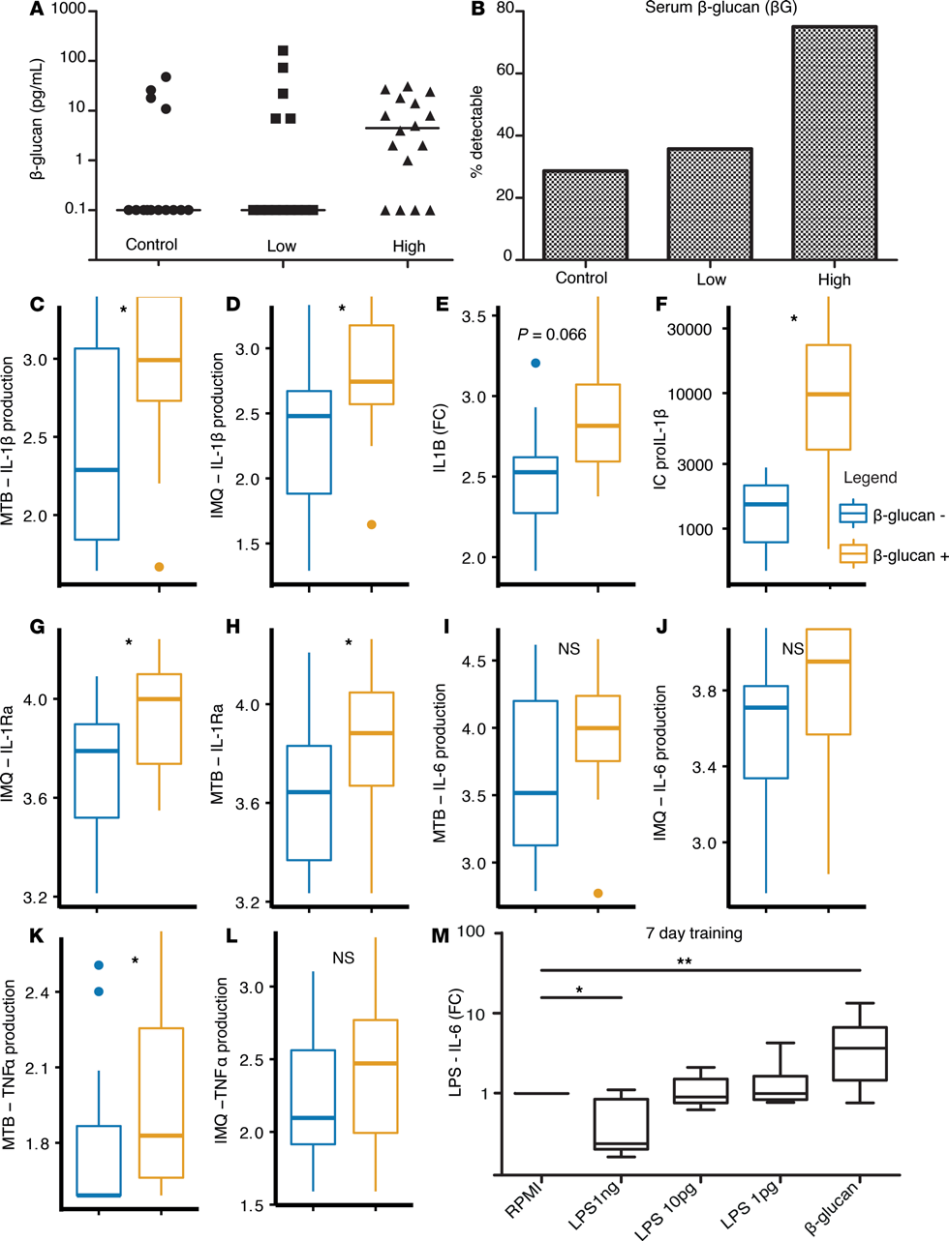
β-Glucan induces a proinflammatory phenotype in monocytes. (**A**) β-Glucan in serum stratified by HCs (control) and PLHIV with low or high initial IL-1β response. (**B**) Percentage of detectable levels of β-glucan in serum, stratified by control and PLHIV with high and low initial IL-1β response. (**C** and **D**) IL-1β production after 24 hours’ stimulation with imiquimod (**C**) (1 μg/mL) or *Mycobacterium tuberculosis* (Mtb) (**D**) (1 μg/mL) in PLHIV stratified by detectable β-glucan levels. (**E**) *IL1B* gene expression after imiquimod stimulation depicted as FC from medium (RPMI). (**F**) Intracellular levels of pro–IL-1β protein after imiquimod stimulation. (**G** and **H**) IL-1Ra production after 24 hours’ stimulation with imiquimod (**G**) or Mtb (**H**) in PLHIV stratified by β-glucan. (**I** and **J**) IL-6 production after 24 hours’ stimulation with imiquimod (**I**) or Mtb (**J**) in PLHIV stratified by β-glucan. (**K** and **L**) TNF-α production after 24 hours’ stimulation with imiquimod (**K**) or Mtb (**L**) in PLHIV stratified by β-glucan. (**A**–**L**) PLHIV, *n* = 28; HCs, *n* = 14. All box plots are depicted according to Tukey; median (line), IQR (edge of box plot), range (whiskers), and outliers 3 times IQR are depicted as dots. Data were analyzed using 2-tailed Student’s *t* test after log10 transformation. (**M**) Initial training with LPS, β-glucan (10 μg/mL), or medium only (RPMI plus 10% serum) was performed for 24 hours on day 1. Thereafter, a 5-day resting period in medium only (supplemented by 10% serum); on day 6, adherent monocytes were restimulated with LPS 10 ng/mL. IL-6 was measured in the supernatant and FC from training with medium only are depicted. Data are from 3 separate experiments (*n* = 9). Data were analyzed using Wilcoxon matched pairs signed-rank test. *P < 0.05 **P < 0.01. IMQ, imiquimod; FC, fold change.

Increased intestinal translocation may also increase circulating concentrations of β-glucan, which is a well-known inducer of trained immunity (28). We therefore determined serum β-glucan concentrations in the subsequent cohort of 28 PLHIV and 12 controls. Compared with controls (4 of 14, 29%), a significantly higher proportion of PLHIV (16 of 28, 57%), and especially those in the high IL-1β–producer group (11 of 15, 73%), had detectable β-glucan in serum (Figure 8, A and B). Individuals with detectable serum β-glucan had elevated IL-1β production (Figure 8, C and D) and showed increased *IL1B* gene expression (Figure 8E) and increased intracellular pro–IL-1β upon stimulation (Figure 8F). Concurrently, IL-1Ra production was increased in PLHIV with detectable β-glucan concentrations (Figure 8G). A similar, nonsignificant trend was observed for IL-6 and TNF-α responses (Figure 8, H–L). Next, using a well-established in vitro training protocol of trained immunity (29), we confirmed that prestimulation of adherent monocytes with β-glucan resulted in a pronounced enhancement of IL-6 release upon restimulation with LPS on day 6 (Figure 8M). We also investigated the effects of prestimulation with LPS. As previously reported, prestimulation with LPS induced immune tolerance (Figure 8D) (15). Taken together, these findings suggest that circulating β-glucan was a possible driver of the proinflammatory immune phenotype of monocytes in PLHIV.

## Discussion

In this study, we investigated the functional phenotype of circulating immune cells in a cohort of PLHIV on stable cART. We show that PLHIV exhibited a sustained elevation in monocyte cytokine responsiveness, whereas lymphocyte-derived cytokine responses were mostly unaffected. Our immune profiling data, which included transcriptome analysis of circulating monocytes, further showed an increased expression and activation of the IL-1β pathway. HIV infection is associated with increased microbial translocation (9), and we identified a role for elevated circulating β-glucan concentrations in the altered inflammatory monocyte phenotype in PLHIV.

These data corroborate findings from earlier, smaller studies in PLHIV, which reported an increased production of proinflammatory cytokines to stimulation with ligands of TLR4 (30, 31) or TLR7 (32, 33) or *M*. *tuberculosis* (34). The increased monocyte-derived cytokine responsiveness in our PLHIV cohort was associated with increased plasma concentrations of different circulating inflammatory markers, such as hsCRP, sCD14, IL-18, and IL-6, suggesting a functional link between changes in the innate immune profile and systemic inflammation in PLHIV. Remarkably, HIV infection had little effect on lymphocyte-derived cytokine responses, despite the fact that alterations in T cell immune phenotype have been described in long-term virally suppressed PLHIV (35, 36). Previous research in HFGP cohorts of HIV-uninfected individuals showed that age, environmental, microbial, and genetic factors have a clear impact on the function of circulating T cells (21–23, 37).

The observed intrinsic proinflammatory phenotype of monocytes in PLHIV was stable over time and associated with upregulation of proinflammatory pathways, most notably the IL-1β pathway. This corroborates a recent study showing a pronounced proinflammatory phenotype, including upregulation of *IL1B*, of monocyte-derived macrophages in PLHIV (38). Long-term epigenetic reprogramming of monocytes in response to microbes has been termed trained immunity. This functional adaptation of monocytes enables a greater response when subjected to a secondary stimulus as a protective mechanism against a secondary infection (14–16). Nonetheless, a trained immunity phenotype of monocytes has also been related to conditions such as CVDs and diabetes (39–42). In PLHIV, altered epigenetic profiles such as DNA methylation have been reported (43–45). Specific DNA methylation patterns in PLHIV were associated with progressive aging and non-AIDS–related comorbidities, such as insulin resistance, neurocognitive disorders, and chronic kidney disease (17, 46–48). Interestingly, HIV/SIV DNA vaccination was recently shown to induce a trained immunity phenotype in vivo through upregulation of IL-1β–related genes (49). This upregulation correlated with protection against subsequent SIV infection in macaques. Furthermore, HIV-1 itself has also been shown to increase IL-6 production via epigenetic modification of STAT3 in microglial cells (50).

Although epigenetic modification has been shown to underlie the trained immunity phenotype, we did not determine epigenetic changes in the current study. Future studies are warranted to investigate the epigenetic processes underlying the trained immunity phenotype reported here.

Another question concerned the identification of the possible mechanisms responsible for the observed changes in innate immune responses. CMV (10), microbial translocation (9), and HIV reservoir (11) have each been related to persistent inflammation in PLHIV. However, our data did not show a positive correlation between monocyte responsiveness and CMV seropositivity, levels of iFABP (microbial translocation marker), or parameters of the HIV reservoir. Although we do not exclude a possible accessory role for these markers of microbial exposure, we did find an association of circulating β-glucan concentrations and IL-1β responsiveness. β-Glucan is a component of the cell wall of fungi and is known to be a strong inducer of trained immunity (16). Furthermore, IL-1β signaling is reported to be important in β-glucan–induced trained immunity in vivo (51). Our present data corroborate earlier reports that increased circulating β-glucan concentrations can be found in virally suppressed PLHIV (28, 52, 53). Acute HIV infection leads to an early and pronounced loss of mucosal Th17 CD4^+^ T cells (54). This cellular compartment is critical for regulation of mucosal host defense against *Candida* and regulation of epithelial cell permeability (55). Increased levels of other microbial products, including LPS, have also been reported in PLHIV (28, 56). However, in contrast to β-glucan, LPS generally induces immune tolerance, even at low concentrations (15, 16), a notion we confirmed in vitro. This notion is supported by our observation that microbial integrity, as measured by iFABP, is associated with decreased monocyte responsiveness. An HIV infection is associated with alterations in the bacterial gut microbiome composition and these changes play a role in the incidence of comorbidities and inflammation (2, 57, 58). Although studies on the mycobiome in PLHIV are limited, fungal dysbiosis with a high prevalence of *Candida* species has been found in stool samples of PLHIV (59). Furthermore, our data showed lower IL-6 and higher IL-10 production by monocytes upon *Candida* stimulation in PLHIV compared with uninfected controls, suggesting an altered fungal immune response. This defective immunity could contribute to fungal dysbiosis in virally suppressed PLHIV. Hence, our present data support the need for future studies exploring the role of the mycobiome in persistent inflammation in PLHIV, as well as possible strategies to reduce β-glucan exposure.

Reducing inflammation is considered an attractive therapeutic target to reduce the burden of CVDs. The Canakinumab Anti-Inflammatory Thrombosis Outcome Study trial was carried out in HIV-uninfected subjects who were at risk for a cardiovascular event. The trial revealed that specific neutralization of IL‑1β reduced the incidence of subsequent cardiovascular events and death from lung cancer (60, 61). Similarly, the antiinflammatory drug colchicine reduced cardiovascular events in patients with coronary disease (62). In contrast, the immunosuppressive drug methotrexate did not reduce CVD, underlining the importance of IL-1β in CVD (63). Recent findings that canakinumab reduced atherosclerotic inflammation in PLHIV at high risk for CVD are therefore promising (64) and support the importance of IL-1β pathway in HIV. Orally administrated drugs that inhibit the IL-1β pathway, such as inflammasome inhibitors, may form an attractive alternative (65). This also applies to epigenetics modifying drugs, such as histone deacetylase inhibitors. These drugs have been shown to reduce CRP concentrations, IL-1β expression in PBMCs (66), and IL-1β production to LPS stimulation in whole blood from PLHIV (67). Taken together, our present data support the use of interventions targeting the IL-1β pathway or reduce β-glucan exposure as an adjunctive therapy to reduce non-AIDS–related comorbidities.

A particular strength of our study is the in-depth functional analysis of adaptive and innate immune cell function in a relatively large cohort of PLHIV. The same HFGP approach was successfully used to investigate genetic, environmental, and microbial factors influencing the immune system in HIV-uninfected individuals (21–23, 37). Limitations of our study are that the age and sex distribution of the PLHIV and control cohorts differed. All analyses were therefore adjusted for these differences using multivariate analyses with sufficient sample size. Moreover, the primary findings were confirmed when we resampled a selection of age- and sex-matched PLHIV and controls. However, we could not correct for men who have sex with men, as this information was not available for the control group (68). Second, the nature of our cross-sectional study does not allow us to draw strong causal inferences, for example, on the role of HIV or β-glucan in the observed immune changes. Third, the heterogeneity and activity of the HIV reservoir is not fully determined by HIV CA-DNA and HIV CA-RNA measurement only; residual viremia with single-copy assay could quantify recent HIV activity. Fourth, recently, monocyte alterations in PLHIV were linked to cART-induced oxidative stress (69). The enrollment in our cohort was limited to virally suppressed PLHIV on cART and, therefore, a possible independent effect of cART on immune responses could not be assessed. However, an effect of different cART regimens appears unlikely because no differences in cytokine responses were found across the different cART regimens. Finally, due to limited inclusion of women in our cohort, generalizability to women or sex-specific analyses were not feasible.

In conclusion, we found that PLHIV on stable cART exhibit a marked and long-lasting increase in the production of IL-1β with subsequent downstream monocyte-derived cytokines, suggesting a trained immunity phenotype of monocytes. Increased translocation of β-glucan from the gastrointestinal tract was identified as a possible inducer of this innate immune phenotype. Our findings provide mechanistic insights and shed new light on the usefulness of interventions targeting the innate immune system, as well as the gut mycobiome, as an adjunctive therapy to reduce non-AIDS–related comorbidities.

## Methods

### Study population.

A total of 211 PLHIV were recruited from the HIV clinic of the Radboud University Medical Center between December 2015 and February 2017, with a follow-up measurement in 2018. Caucasian individuals who were 18 years of age or older, were on cART for more than 6 months with an HIV-RNA load greater than or equal to 200 copies/mL, and showed no signs of opportunistic infections or active hepatitis B/C were included. The control group consisted of 56 healthy individuals not using any medication at inclusion. Inclusion, sampling, and sample processing of both cohorts were conducted simultaneously, and uninfected controls were sampled every 3 months during the inclusion of PLHIV. In the follow-up measurement, we resampled 28 PLHIV, men older than 45 years of age, after more than 1 year and stratified them on the basis of their initial IL-1β production capacity in a group of low IL-1β–producers (lowest quartile) and high IL-1β–producers (highest quartile). A control group of 14 age- and sex-matched healthy controls was also included. These groups were used for monocyte-only RNA expression and β-glucan measurements. All experiments were performed by the same personnel using the HFGP methodologies (http://www.humanfunctionalgenomics.org) (19). General information from all participants was recorded in an electronic case report form (CastorEDC). Clinical data were extracted from the electronic hospital information system and the Stichting HIV Monitoring registry.

### Ex vivo PBMC and monocyte stimulation.

Venous blood was collected in sterile 10 mL EDTA and 8 mL serum BD Vacutainer tubes (Becton Dickinson) and processed within 1–4 hours. Isolation of PBMCs was performed on freshly collected blood by density centrifugation over Ficoll-Paque (VWR) as described previously (70). Monocytes were isolated by magnetic-activated cell sorting using negative bead selection with the Pan Monocyte Isolation Kit (Miltenyi Biotec) according to manufacturer’s instructions. Cell counts, cell purity, and composition were evaluated by XN-450 hematology analyzer (Sysmex Corporation). Fresh isolated cells (monocytes 1 × 10^5^ cells per well, PBMCs 5 × 10^5^ cells per well) were incubated with different bacterial, fungal, and viral stimuli (Supplemental Table 2) at 37°C and 5% CO_2_ for either 24 hours or 7 days. For the 7-day stimulation, 10% human pooled serum was added to the wells. IL-1β, IL-6, IL-1Ra, IL-10, and TNF-α were determined in the supernatants of the 24-hour PBMC or monocyte stimulation experiments, using ELISAs (Duoset ELISA, R&D Systems). IL-17, IL-22, and IFN-γ were measured after the 7-day stimulation of PBMCs (PeliKine Compact or R&D Systems). For intracellular cytokine measurements of active IL-1β and pro–IL-1β (Quantikine, R&D Systems), cell pellets were lysed using Triton X-100 (MilliporeSigma).

### Measurements of plasma markers and immunophenotyping.

iFABP, a marker of enterocyte damage, resistin, adiponectin, leptin, IL-18BP, IL-18, hsCRP, sCD14, and sCD163 were measured using ELISA (Duoset or Quantikine, R&D Systems). IL-6, TNF-α, IL-10, and IL-1Ra were measured using SimplePlex Cartridges (Protein Simple). Levels of 1,3-β-D-glucan (β-glucan) were measured with Fungitell (Associates of Cape Cod) and CMV IgG by ELISA (Genway Biotech). All assays were performed according to manufacturers’ recommendations, and samples of the different cohorts were measured simultaneously in the same plates or SimplePlex Cartridges. Monocyte subsets were measured in whole blood on a Navios flow cytometer (Beckman Coulter) as described elsewhere. Antibodies used are listed in Supplemental Table 3 (22).

### RNA extraction and gene expression.

For qPCR analyses, total RNA was extracted from monocyte cell pellets (5 × 10^5^ cells) collected in TRIzol (Life Technologies) according to the manufacturer’s instructions. Subsequently, cDNA was synthesized using iScript reverse transcriptase according to manufacturer’s instructions (Invitrogen) with 500 ng RNA input in a 10 μL reaction mix. Relative expression of genes *NLRP3*, *TNF*, *IL6*, and *IL1B* was measured using SYBR Green assays (Invitrogen) on an Applied Biosciences Step-One PLUS qPCR machine (Thermo Fisher Scientific) withr eaction volumes of 10 μL with 2 μL cDNA input. Normalization was performed using ΔΔCt based on 2 (*18S* and *B2M*) reference genes that were stable after stimulation. Cycling conditions were 95°C for 10 minutes, 40 cycles at 95°C for 15 seconds, 60°C for 1 minute, followed by dissociation curve analysis. Primers were designed to be intron spanning and are listed in Supplemental Table 4.

For transcriptome analysis by RNA-Seq, total RNA was extracted from monocytes (5 × 10^5^) using the QIAGEN RNeasy RNA extraction kit, using on-column DNase treatment. Next, ribosomal RNA removal and library preparation for next-generation RNA-Seq were achieved utilizing KAPA RNA HyperPrep Kit with RiboErase (Roche), following the manufacturer’s protocol. The integrity and quality of prepared libraries were assessed using Agilent 2100 Bioanalyzer. Sequencing was performed using Illumina NextSeq 500 machine in a paired-end sequencing fashion. Sequencing reads obtained from RNA-Seq measurement were aligned to the hg38 human genome reference using STAR (71). A lower cutoff of average 50 reads among all samples was used to designate a gene as being expressed in our cohort. R software and DESeq2 differential analysis package (72) were used to normalize and assess differentially expressed genes, utilizing FDR 5% and log2(fold change) greater than 1 as the significance cutoff. The network of genes connected to their corresponding gene ontology was generated using cytoscape (73). The processed RNA-Seq data including normalized read counts can be accessed via the National Center for Biotechnology Information’s Gene Expression Omnibus database (GSE160184).

### HIV reservoir quantification.

*HIV-1* CA-DNA and CA-RNA were measured in triplicate by droplet digital PCR (ddPCR QX200 — Bio-Rad) (Supplemental Table 3) in CD4^+^ T cells isolated using EasySep Human CD4+ T Cell Isolation Kit (STEMCELL Technologies) as previously described (74). Briefly, genomic DNA was extracted using the DNeasy Blood & Tissue Kit (QIAGEN) according to the manufacturer’s protocol, with an additional step of adding 75 μL elution buffer on the column heated at 56°C for 10 minutes. CA-RNA was extracted using the Innuprep RNA kit (Westburg) with 30 μL elution buffer. Total RNA was reversely transcribed to cDNA by qScript cDNA SuperMix according to manufacturer’s protocol (Quantabio). Before PCR amplification, genomic DNA was restricted by EcoRI (Promega), and cycling conditions were implemented as described previously (74). Total *HIV-1* DNA measurements were normalized by measuring the reference gene RPP30 (Supplemental Table 4) in duplicate by ddPCR and expressed per million PBMCs. CA-RNA was normalized using 3 reference genes per patient (*B2M*, *ACTB*, and *GADPH*), which were measured with LightCycler 480 SYBR Green Master Mix (Bio-Rad). *HIV-1* RNA copies were divided by the geometric mean of the reference genes and expressed per million PBMCs. Droplet classification and absolute quantification were performed using the ddpcRquant analysis tool with standard settings (75).

### Statistics.

Depending on normality, log10- or inverse rank-based transformation was applied. Values outside the assay quantification limits were imputed at the respective limit. Parameters for which one of the detection limits contained greater than 50% of the measurements were excluded. Comparisons in baseline characteristics between groups were made using 2-tailed Student’s *t* test or Mann-Whitney *U* test depending on data distribution. Differences in noncontinuous data were analyzed by the Pearson’s χ^2^ test or Fisher’s exact test in case of expected counts less than 5. Data were analyzed using a linear regression model. The crude model included an adjustment for sampling time and the primary analyses included age, sex, and seasonality as covariates. A *P* value less than 0.05 was considered statistically significant, and correction for multiple testing was applied using the Benjamini-Hochberg method (FDR correction). Data were analyzed using R.

### Study approval.

The study protocol was approved by the Medical Ethical Review Committee region Arnhem-Nijmegen (CMO2012-550), and experiments were conducted in accordance with the principles of the Declaration of Helsinki. Written informed consent was obtained from all participants.

## Author contributions

WAVDH, LVDW, QDM, MGN, LABJ, and AJAMVDV designed the study. WAVDH, LVDW, and MJ recruited and included the participants. WAVDH, LVDW, MJ, WT, FK, and SR performed the laboratory experiments. WAVDH, RTH, FK, and LVDW analyzed the data and interpreted the data together with QDM, AJAMVDV, MGN, CAD, LV, PEV, HJPMK, HGS, IJ, and JVL. WAVDH, LVDW, AJAMVDV, and QDM wrote the manuscript. All authors have read and contributed significantly to the final manuscript. The co–first authors are listed alphabetically. QDM was listed last in the author list as he supervised the final stages of the project.

## Supplementary Material

Supplemental data

Supplemental Data Set 1

Supplemental Table 5

## Figures and Tables

**Table 1:**
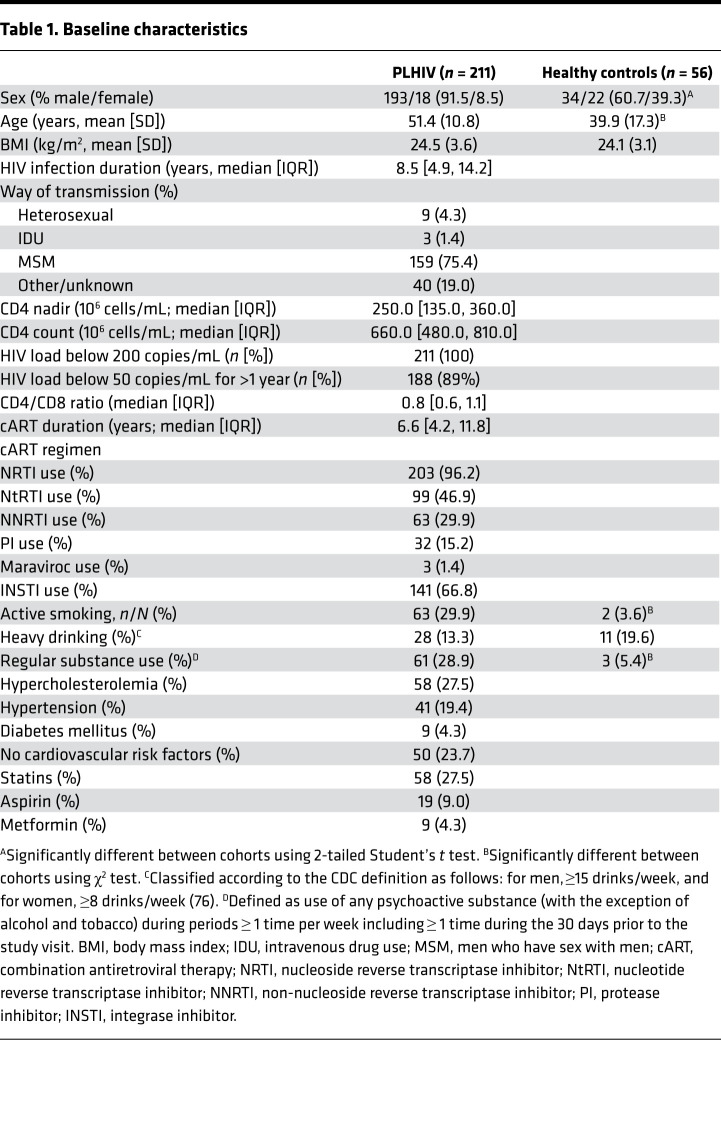
Baseline characteristics

## Version 1 02/25/2021

In-Press Preview

## Version 2 04/08/2021

Electronic publication
