# Supplementary material for: Chronic HIV infection induces transcriptional and functional reprogramming of innate immune cells
Source: JCI Insight. 2021 Apr 8;6(7):e145928. doi: 10.1172/jci.insight.145928 (PMC8119206; doi:10.1172/jci.insight.145928)
Supplement: Supplemental data [file jciinsight-6-145928-s047.pdf]

Supplemental table S1 Baseline characteristics validation cohort

	HIV (n=28)	HC (n=14)
**Male sex, no. (%)**	28 (100)	14 (100)
**Age, median (IQR) years**	56.3 (9.6)	64.3 (12.5)
**cART regimen, no. (%)**		
**NNRTI**	6 (21.4)	-
**PI**	2 (7.1)	-
**Integrase inhibitor**	20 (71.4)	-
**DTG**	16 (57.1)	-
**ELV**	1 (3.6)	-
**RAL**	3 (10.7)	-

Supplementary table S2 Stimulations scheme for the ex vivo cytokine production assays.

Stimulus	Final Concentration	Strain/Manufacture
	**Stimulation of PBMCs for 24 hours**	
**LPS**	1 and 100 ng/mL	E.coli/Sigma
**Pam3Cys**	10 μg/mL	EMC microcollections
**RPMI (+ serum**	10% human serum	Life Technologies
**Poly I:C**	100 μg/mL	Sas Invivogen
**Imiquimod (TLR7 ligand)**	5 ug/mL	Invivogen
**Influenza + serum**	1.25 x 10^6 PFU/mL + 10% human serum	H1N1 (in house)
**Mycobacterium tuberculosis**	1 μg/mL	H37Rv (in house)
**E.Coli**	1 x 10^6/mL	ATCC 35218 (in house)
**Candida albicans conidiae**	1 x 10^6/mL	UC820 (in house)
**Candida albicans hyphae**	1 x 10^6/mL	UC820 (in house)
**Staphylococcus aureus**	1 x 10^6/mL	ATCC 29213 (in house)
**Cryptococcus gattii + serum**	1 x 10^7/mL + 10% human serum	A1M-R265, AFLP type 6 (clinical isolate)
**Streptococcus pneumonia**	1 x 10^7/mL	TIGR4
	**Stimulation of PBMCs for 7 days***	
**RPMI + serum**	10% human serum	Life technologies
**Staphylococcus aureus**	1 x 10^6/mL	
**Cryptococcus gattii**	1 x 10^7/mL	A1M-R265, AFLP type 6 (clinical isolate)
**Candida albicans conidiae**	1 x 10^6/mL	UC820 (in house)
**Candida albicans hyphae**	1 x 10^6/mL	UC820 (in house)
**Streptococcus pneumonia**	1 x 10^6/mL	TIGR4
**Mycobacterium tuberculosis**	1 μg/mL	H37Rv (in house)
**Imiquimod (TLR7 ligand)**	2.5 ug/mL	Invivogen

PBMCs: peripheral blood mononuclear cells; LPS: lipopolysaccharide; TLR: Toll-like receptor.

**All 7-day PBMC stimulation experiments were supplemented with 10% human pool serum.*

Supplemental table S3 Antibodies for immunophenotyping

**Fluorochrome**	FITC	PE	ECD	PE-Cy5.5	PC7	APC	APC-AF700	APC-AF750	PB	KO
**mAb**	CD16	HLA-DR	CD14	CD4	CD25	CD56	CD8	CD19	CD3	CD45
**Distributor**	BC	BC	BC	BC	BD	BC	BC	BC	BC	BC

10-color flow cytometry panel. Samples were analyzed by a 3-laser Navios (BC).
mAb: monoclonal antibody; BC: Beckman Coulter, CA, USA, BD: BD biosciences, CA, USA

Supplemental table S4 Primers and probes

**Gene**	**Fw/Rv/Pr**	**Sequence (5>3)**
**NLRP3**	forward	GATCTTCGCTGCGATCAACA
	reverse	GGGATTCGAAACACGTGCATTA
18S	forward	GATGGGCGGCGGAAAATAG
	reverse	GCGTGGATTCTGCATAATGGT
B2M	forward	ATGAGTATGCCTGCCGTGTG
	reverse	CCAAATGCGGCATCTTCAAAC
RPL37A	forward	ATTGAAATCAGCCAGCACG
	reverse	AGGAACCACAGTGCCAGAT
TNFa	forward	CCTGCTGCACTTTGGAGTGA
	reverse	GAGGGTTTGCTACAACATGGG
IL6	forward	GGCACTGGCAGAAAACAACC
	reverse	GCAAGTCTCCTCATTGAATCC
IL1b	forward	ACAGATGAAGTGCTCCTTCCAG
	reverse	CATGGCCACAACAACTGACG
HIV-1 DNA	forward	GCCTCAATAAAGCTTGCC
	reverse	GGCGCCACTGCTAGAGATTTT
	probe	AAGTRGTGTGTGCCC
RPP30	forward	AGATTTGGACCTGCGAGCG
	reverse	GAGCGGCTGTCTCCACAAGT
	probe	TTCTGACCTGAAGGCTCTGCGCG

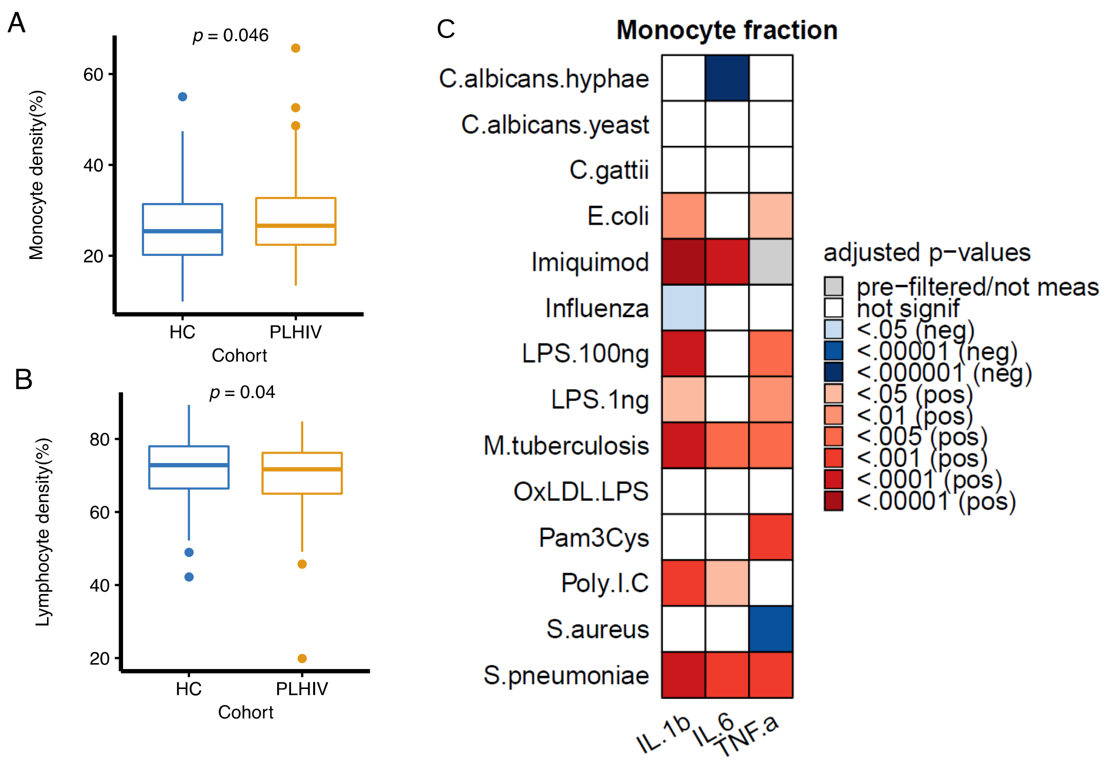

Supplemental figure S1: A-B Composition of PBMCs determined by haematology analyzer. A: depicts percentage (%) of monocytes in PBMC. B: lymphocyte percentage of total PBMCs. C: Adjusted P-value are depicted from a corrected model including age, sex, seasonality and monocyte fraction (of PMBCs) as covariates. Red depicts significantly higher in PLHIV, blue depicts lower in PLHIV compared to controls. All p-values are FDR corrected.

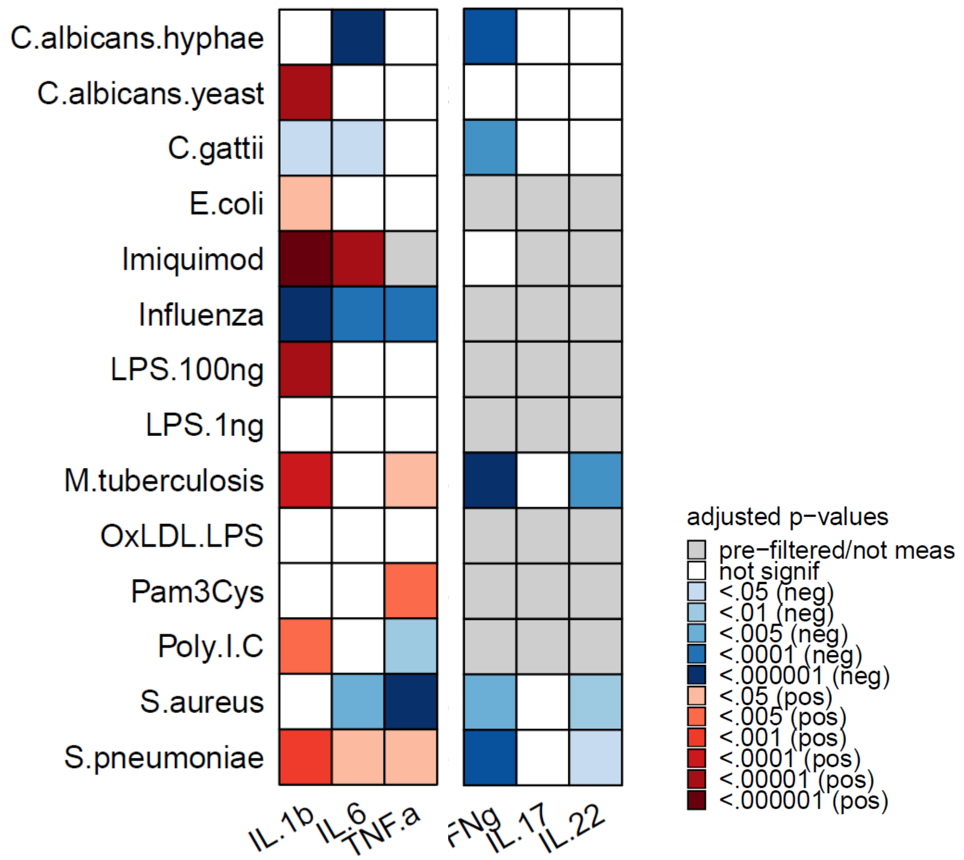

Supplemental Figure S2; Crude values 24h and 7 days cytokine production. Circulating factors in PLHIV and uninfected controls. Crude model is linear regression after inverse rank-based transformation. Corrected model included age, sex, and seasonality as covariates. Red depicts the marker is significantly increased in PLHIV, blue depicts the marker is decreased in PLHIV compared to controls. All p-values are FDR corrected.

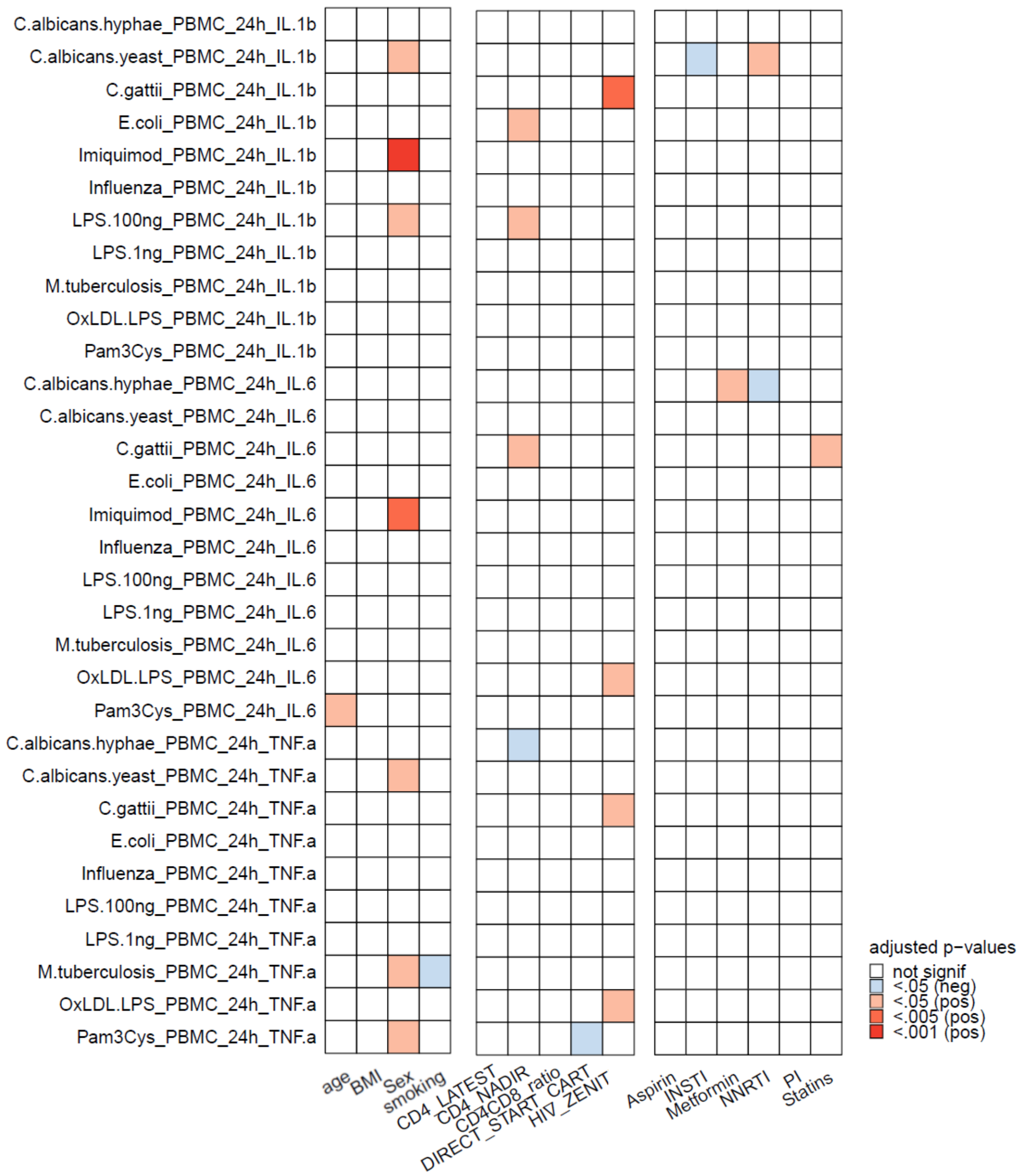

Supplemental figure S3: Adjusted P-value are depicted from a corrected linear regression model including age, sex, seasonality as covariates. Red depicts a significant positive correlation, blue depicts negative correlation. All p-values are FDR corrected.

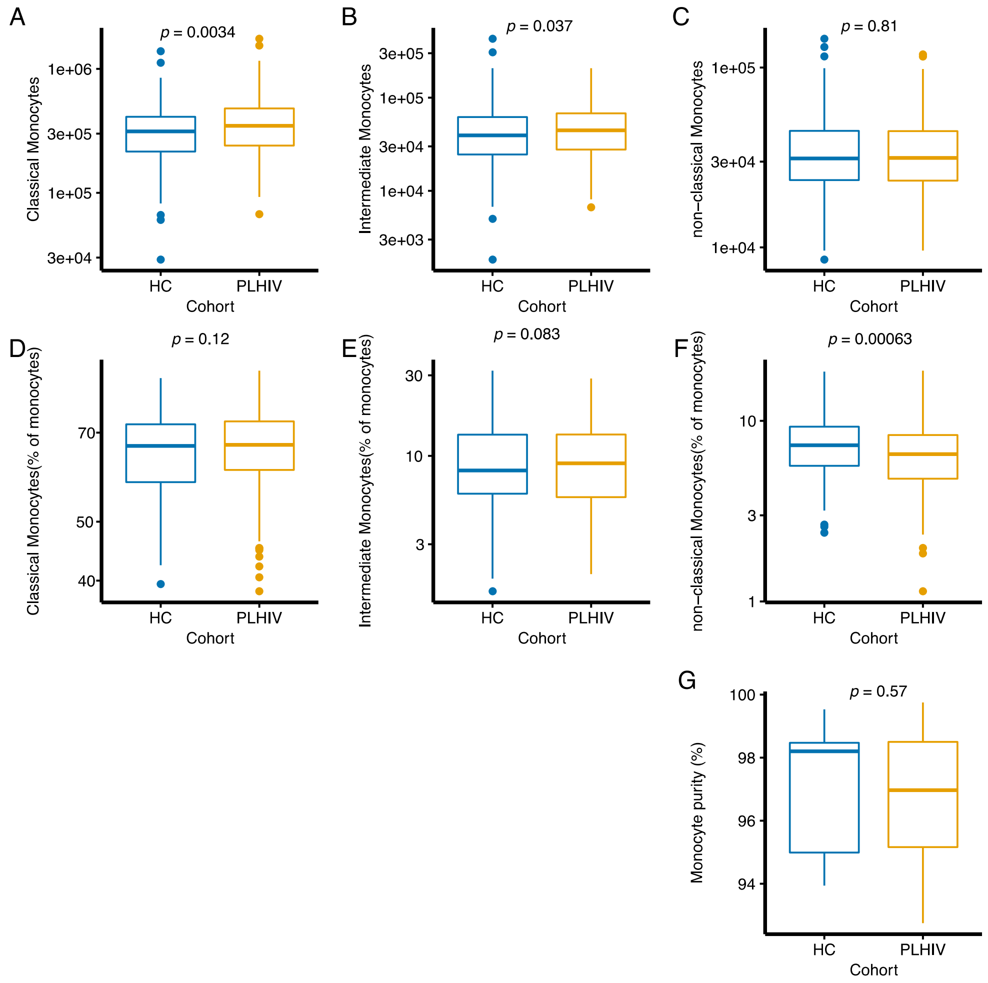

Supplemental figure S4 Monocytes. A-C Circulating monocyte subset concentrations determined by immunophenotyping. Classical monocytes(A): CD14++CD16-, Intermediate(B): CD14++CD16+, non-classical(C): CD14+CD16+. D-F subsets are depicted as % of total monocytes. G: Monocyte purity after CD14-negative selection magnetic bead isolation determined by Sysmex XN-450 hematology analyzer.

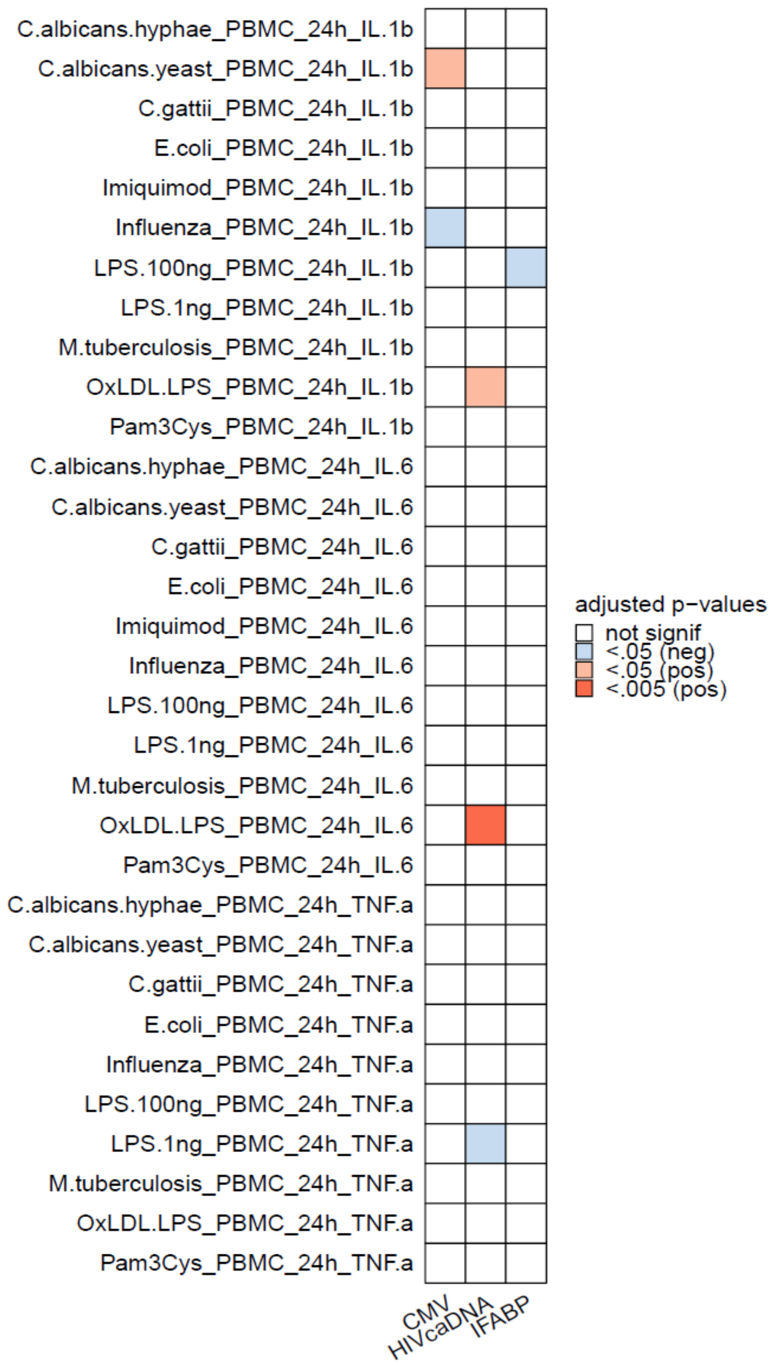

Supplemental figure S5: HIV reservoir, CMV seropositivity and microbial integrity. P-value are depicted from linear regression. Red depicts a significant positive correlation, blue depicts negative correlation. All p-values are FDR corrected.
